# 709 Augmented Renal Clearance (ARC) is Highly Prevalent in Critically Ill Burn Patients

**DOI:** 10.1093/jbcr/irad045.185

**Published:** 2023-05-15

**Authors:** Christopher O'Neil, Walter Ramsey, Brianna Cohen, Nicole Lyons, Nicholas Namias, Shevonne Satahoo, Joyce Kaufman, Louis Pizano, Carl Schulman, Kenneth Proctor, Edward Lineen

**Affiliations:** DeWitt Daughtry Family Department of Surgery, University of Miami, Miami, Florida; DeWitt Daughtry Family Department of Surgery, University of Miami, Miami, Florida; DeWitt Daughtry Family Department of Surgery, University of Miami, Miami, Florida; DeWitt Daughtry Family Department of Surgery, University of Miami, Miami, Florida; DeWitt Daughtry Family Department of Surgery, University of Miami, Miami, Florida; University of Miami, Miami, Florida; University of Miami, Miami, Florida; University of Miami, Miami, Florida; University of Miami-Jackson Memorial Hospital, Miami, Florida; DeWitt Daughtry Family Department of Surgery, University of Miami, Miami, Florida; DeWitt Daughtry Family Department of Surgery, University of Miami, Miami, Florida; DeWitt Daughtry Family Department of Surgery, University of Miami, Miami, Florida; DeWitt Daughtry Family Department of Surgery, University of Miami, Miami, Florida; DeWitt Daughtry Family Department of Surgery, University of Miami, Miami, Florida; DeWitt Daughtry Family Department of Surgery, University of Miami, Miami, Florida; DeWitt Daughtry Family Department of Surgery, University of Miami, Miami, Florida; University of Miami, Miami, Florida; University of Miami, Miami, Florida; University of Miami, Miami, Florida; University of Miami-Jackson Memorial Hospital, Miami, Florida; DeWitt Daughtry Family Department of Surgery, University of Miami, Miami, Florida; DeWitt Daughtry Family Department of Surgery, University of Miami, Miami, Florida; DeWitt Daughtry Family Department of Surgery, University of Miami, Miami, Florida; DeWitt Daughtry Family Department of Surgery, University of Miami, Miami, Florida; DeWitt Daughtry Family Department of Surgery, University of Miami, Miami, Florida; DeWitt Daughtry Family Department of Surgery, University of Miami, Miami, Florida; DeWitt Daughtry Family Department of Surgery, University of Miami, Miami, Florida; University of Miami, Miami, Florida; University of Miami, Miami, Florida; University of Miami, Miami, Florida; University of Miami-Jackson Memorial Hospital, Miami, Florida; DeWitt Daughtry Family Department of Surgery, University of Miami, Miami, Florida; DeWitt Daughtry Family Department of Surgery, University of Miami, Miami, Florida; DeWitt Daughtry Family Department of Surgery, University of Miami, Miami, Florida; DeWitt Daughtry Family Department of Surgery, University of Miami, Miami, Florida; DeWitt Daughtry Family Department of Surgery, University of Miami, Miami, Florida; DeWitt Daughtry Family Department of Surgery, University of Miami, Miami, Florida; DeWitt Daughtry Family Department of Surgery, University of Miami, Miami, Florida; University of Miami, Miami, Florida; University of Miami, Miami, Florida; University of Miami, Miami, Florida; University of Miami-Jackson Memorial Hospital, Miami, Florida; DeWitt Daughtry Family Department of Surgery, University of Miami, Miami, Florida; DeWitt Daughtry Family Department of Surgery, University of Miami, Miami, Florida; DeWitt Daughtry Family Department of Surgery, University of Miami, Miami, Florida; DeWitt Daughtry Family Department of Surgery, University of Miami, Miami, Florida; DeWitt Daughtry Family Department of Surgery, University of Miami, Miami, Florida; DeWitt Daughtry Family Department of Surgery, University of Miami, Miami, Florida; DeWitt Daughtry Family Department of Surgery, University of Miami, Miami, Florida; University of Miami, Miami, Florida; University of Miami, Miami, Florida; University of Miami, Miami, Florida; University of Miami-Jackson Memorial Hospital, Miami, Florida; DeWitt Daughtry Family Department of Surgery, University of Miami, Miami, Florida; DeWitt Daughtry Family Department of Surgery, University of Miami, Miami, Florida; DeWitt Daughtry Family Department of Surgery, University of Miami, Miami, Florida; DeWitt Daughtry Family Department of Surgery, University of Miami, Miami, Florida; DeWitt Daughtry Family Department of Surgery, University of Miami, Miami, Florida; DeWitt Daughtry Family Department of Surgery, University of Miami, Miami, Florida; DeWitt Daughtry Family Department of Surgery, University of Miami, Miami, Florida; University of Miami, Miami, Florida; University of Miami, Miami, Florida; University of Miami, Miami, Florida; University of Miami-Jackson Memorial Hospital, Miami, Florida; DeWitt Daughtry Family Department of Surgery, University of Miami, Miami, Florida; DeWitt Daughtry Family Department of Surgery, University of Miami, Miami, Florida; DeWitt Daughtry Family Department of Surgery, University of Miami, Miami, Florida; DeWitt Daughtry Family Department of Surgery, University of Miami, Miami, Florida; DeWitt Daughtry Family Department of Surgery, University of Miami, Miami, Florida; DeWitt Daughtry Family Department of Surgery, University of Miami, Miami, Florida; DeWitt Daughtry Family Department of Surgery, University of Miami, Miami, Florida; University of Miami, Miami, Florida; University of Miami, Miami, Florida; University of Miami, Miami, Florida; University of Miami-Jackson Memorial Hospital, Miami, Florida; DeWitt Daughtry Family Department of Surgery, University of Miami, Miami, Florida; DeWitt Daughtry Family Department of Surgery, University of Miami, Miami, Florida; DeWitt Daughtry Family Department of Surgery, University of Miami, Miami, Florida; DeWitt Daughtry Family Department of Surgery, University of Miami, Miami, Florida; DeWitt Daughtry Family Department of Surgery, University of Miami, Miami, Florida; DeWitt Daughtry Family Department of Surgery, University of Miami, Miami, Florida; DeWitt Daughtry Family Department of Surgery, University of Miami, Miami, Florida; University of Miami, Miami, Florida; University of Miami, Miami, Florida; University of Miami, Miami, Florida; University of Miami-Jackson Memorial Hospital, Miami, Florida; DeWitt Daughtry Family Department of Surgery, University of Miami, Miami, Florida; DeWitt Daughtry Family Department of Surgery, University of Miami, Miami, Florida; DeWitt Daughtry Family Department of Surgery, University of Miami, Miami, Florida; DeWitt Daughtry Family Department of Surgery, University of Miami, Miami, Florida; DeWitt Daughtry Family Department of Surgery, University of Miami, Miami, Florida; DeWitt Daughtry Family Department of Surgery, University of Miami, Miami, Florida; DeWitt Daughtry Family Department of Surgery, University of Miami, Miami, Florida; University of Miami, Miami, Florida; University of Miami, Miami, Florida; University of Miami, Miami, Florida; University of Miami-Jackson Memorial Hospital, Miami, Florida; DeWitt Daughtry Family Department of Surgery, University of Miami, Miami, Florida; DeWitt Daughtry Family Department of Surgery, University of Miami, Miami, Florida; DeWitt Daughtry Family Department of Surgery, University of Miami, Miami, Florida; DeWitt Daughtry Family Department of Surgery, University of Miami, Miami, Florida; DeWitt Daughtry Family Department of Surgery, University of Miami, Miami, Florida; DeWitt Daughtry Family Department of Surgery, University of Miami, Miami, Florida; DeWitt Daughtry Family Department of Surgery, University of Miami, Miami, Florida; University of Miami, Miami, Florida; University of Miami, Miami, Florida; University of Miami, Miami, Florida; University of Miami-Jackson Memorial Hospital, Miami, Florida; DeWitt Daughtry Family Department of Surgery, University of Miami, Miami, Florida; DeWitt Daughtry Family Department of Surgery, University of Miami, Miami, Florida; DeWitt Daughtry Family Department of Surgery, University of Miami, Miami, Florida; DeWitt Daughtry Family Department of Surgery, University of Miami, Miami, Florida; DeWitt Daughtry Family Department of Surgery, University of Miami, Miami, Florida; DeWitt Daughtry Family Department of Surgery, University of Miami, Miami, Florida; DeWitt Daughtry Family Department of Surgery, University of Miami, Miami, Florida; University of Miami, Miami, Florida; University of Miami, Miami, Florida; University of Miami, Miami, Florida; University of Miami-Jackson Memorial Hospital, Miami, Florida; DeWitt Daughtry Family Department of Surgery, University of Miami, Miami, Florida; DeWitt Daughtry Family Department of Surgery, University of Miami, Miami, Florida

## Abstract

**Introduction:**

Augmented renal clearance (ARC) has been reported to occur across many critical illnesses but has not been evaluated in critically ill burn patients. The impact of ARC on clinical outcomes within this population remains unknown. We hypothesize that ARC is prevalent in critically ill burn patients and is associated with improved survival.

**Methods:**

We retrospectively reviewed a prospectively-maintained registry of Burn ICU patients from July 2021 – September 2022 at an academic burn center. We included patients in whom 24-hour urine creatinine collection was performed on admission and excluded patients in whom accurate collection was not performed within 48 hours of admission. Creatinine clearance was calculated for all patients who met inclusion criteria. ARC was determined to occur when creatine clearance exceeded 130 mL/min/1.73m^2^. Clinical outcomes were compared between patients with and without ARC.

**Results:**

The analysis included 24 patients (67% male, median age 42 [31-55] years). The median percentage of total body surface area (TBSA) burned was 25 [10-38]. ARC was present in 17 patients (71%). Mean creatinine clearance was 162 ml/min/1.73m^2^ (range 37-313), and 7 patients (29%) had creatinine clearance greater than 200. Complication rates were low and were similar between patients with and without ARC (all p >0.05).

**Conclusions:**

ARC appears to be a common phenomenon among critically injured burn patients. While the presence of ARC could be particularly meaningful in this population of patients that often receives massive volumes of resuscitative fluids, the sample size of our study did not permit the detection of statistically significant differences in outcomes between burn patients with and without ARC. Further work must be undertaken to assess the impact of ARC on fluid resuscitation strategies and medication dosing including that of antibiotics and thromboprophylaxis agents.

**Applicability of Research to Practice:**

Augmented renal clearance (ARC) has never before been characterized in the population of critically injured burn patients. This topic merits further research, as it could have far-reaching clinical impact relating to fluid resuscitation practices and dosing of renally-cleared medications (antibiotics, thromboprophylaxis, etc.).

